# Delivery of anti-cancer and anti-depression doxepin drug by nickel oxide nanoparticles originated from the *Cressa nudicaulis* plant extract

**DOI:** 10.1039/d2ra07545h

**Published:** 2023-04-18

**Authors:** Yan Lu, Mingru Han, Effat Esmaeili Shahri, Sedighe Abbaspour, Reza Tayebee

**Affiliations:** a Department of Pharmacy, Shandong Cancer Hospital and Institute, Shandong First Medical University and Shandong Academy of Medical Sciences No. 440 Jiyan Road Jinan 250117 Shandong China; b School of Pharmaceutical Sciences, Zhengzhou University Zhengzhou 450001 Henan China; c Department of Chemistry, Payame Noor University (PNU) Tehran Iran; d Department of Chemistry, School of Sciences, Hakim Sabzevari University Sabzevar 96179-76487 Iran rtayebee@hsu.ac.ir

## Abstract

In this research, the extract of *Cressa nudicaulis* plant has been used as a natural reducing agent in order to prepare stable nickel oxide nanoparticles (NiO NPs) using an aqueous solution of nickel(ii) nitrate under the sol–gel method. Additionally, NiO NPs were distinguished using FT-IR (Fourier transform infrared spectroscopy), XRD (X-ray diffraction), FESEM (field-emission scanning electron microscopy), EDS (energy-dispersive X-ray spectrometry), TEM (transmission electron microscopy), and UV-Vis (ultraviolet-visible spectroscopy) techniques. The integrated NiO NPs were loaded with doxepin drug as an effective medication for head and neck cancer as well as depression. Then, the ideal loading circumstances such as pH of the medium, response time, and amount of nanoparticles were assessed to attain that pH 6, time 12 h, and nanoparticle amount of 0.02 g are optimal to accomplish the best drug loading of around 68%. The drug release properties of drug-loaded NiO were also investigated at pH 6.5 and 37 °C. This study showed that ∼73% of the loaded drug was released after 80 h. Therefore, the introduced delivery system shows sufficiently long targeted-release properties. Besides, the MTT experiment was utilized to investigate the cytotoxicity of NiO NPs on the human hepatocellular carcinoma cell line Huh-7.

## Abstract 2

*Cressa nudicaulis* is used to prepare NiO NPs. The prepared nanoparticles were approved for doxepin loading. The drug release properties were also investigated at pH 6.5 and 37 °C. An MTT experiment was performed on the human hepatocellular carcinoma cell line Huh-7.

## Graphical abstract

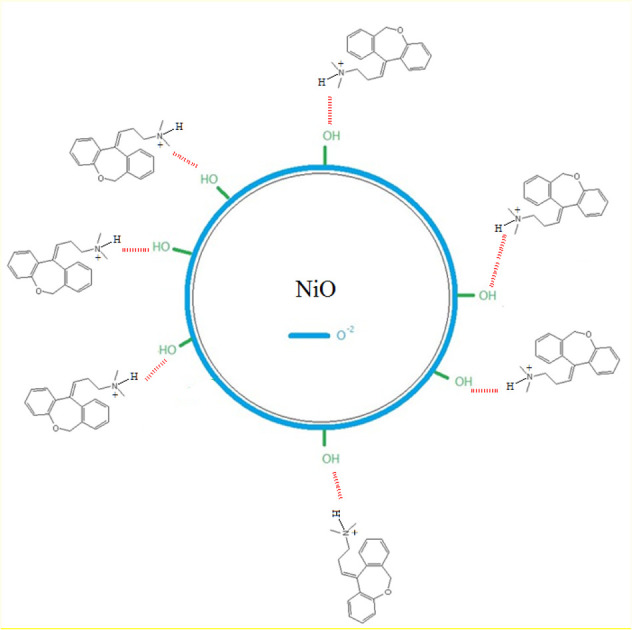

## 1 Introduction

Nanotechnology is an important branch of new sciences corresponding to nanoparticles with sizes below 100 nanometers and accomplishes different designs with tunable sizes using different engineered methods.^1^ The ability to oversee materials at the nanoscale can possibly change medical care and clinical treatment strategies.^2^ Nanoparticles are progressively utilized in the fields of biomolecule architecture, synergistic materials, food, medicine, and material science.^3^ It has been demonstrated that diminishing the size of the bulk materials to the nanosize range can modify their physicochemical properties, making them valuable in an assortment of biomedical applications.^4^ Nanoelectronics, nanomaterials, and nanobiotechnology are the three main branches of nanotechnology.^5^ Metal oxide nanoparticles definitely stand out in light of the fact that they can possibly expand the medication strategies by bringing down the multidrug obstruction and creating more viable and helpful agents.^6^ These nanoparticles offer various advantages in drug delivery, including better bioavailability and biocompatibility toward a specific target, having high surface area, tunable reactivity, and characteristically cytotoxic properties against cancer cells.^7–9^ Therefore, metal oxide nanoparticles have attracted much interest because of their extraordinary potential applications in drug delivery.^10–13^ Currently, nickel oxide nanoparticles have played a significant impact in medical treatments and clinical fields. NiO nanoparticles prompt oxidative pressure in cells that are generally caused by ion migration toward the nuclear cell membrane. Hence, NiO nanoparticles are inclined to be harmful against numerous cancers.^14^ Various strategies have been accounted for the synthesis of these important nanoparticles by utilizing ecologically harmless procedures.^15–17^ Regardless, when contrasted with other green methodologies, plant extracts have arisen as an important strategy since they can be utilized in various settings and require no specific treatments or chemical conditions.^18^ A recent study shows that green synthesized NiO nanoparticles are effective carriers to deliver doxorubicin drug, and also bare nanoparticles have shown anti-cancer activity against the RT4 cancer cell line.^19^

This work planned to develop a green, straightforward, and modest strategy to perform biosynthesis of nickel oxide nanoparticles (NiO NPs) utilizing *Cressa nudicaulis* extract. The as-prepared nanoparticles were then described by FT-IR, XRD, FESEM, EDS, TEM, and UV-Vis. As previously mentioned, doxepin is a unique drug that has a broad range of applications against many cancers, such as blood, lymph system, bladder, breast, stomach, lungs, ovaries, thyroid, nerves, kidneys, and bone cancers. Herein, we attempted to investigate the capacity of the orchestrated NiO NPs in the delivery of this significant drug. Likewise, the impact of pH, response time, and nanoparticle amount were examined on the loading of doxepin. Moreover, the biocompatibility of NiO NPs was inspected against the growth of the Huh-7 cell line, utilizing the MTT assay.

## 2 Materials and methods

### 2.1 Chemicals and apparatus

*Cressa nudicaulis* plant was obtained from Khorasan Razavi, Iran. Doxorubicin (98%) was purchased from Rasta Imen Daroo Organization, and the newly prepared stock solutions were kept at 5 °C. Nickel(ii) nitrate (99.99%), HCl (37%), and NaOH (≥98%) were obtained from Sigma-Aldrich and used without any additional refinement. All of the experiments used water that had been twice refined. Dulbecco's Adjusted Falcon's Medium (DMEM) containing 10% fetal bovine serum (FBS) and (5,4-dimethylthiazole-2-yl)-5,2-diphenyltetrazolium bromide (≥97.5%) were used to test the cytotoxicity by MTT assay. The infrared spectra were recorded using a PerkinElmer FT-IR spectrophotometer between 400 and 4000 cm^−1^. The morphology of the samples was assessed utilizing a ZEISS Organization Sigma VP, Field Emanation Examining Electron Magnifying instrument (FESEM) combined with energy-dispersive spectroscope (EDS). An X'pert Expert diffractometer (PANalytical) was utilized to perform X-ray powder diffraction (XRD) utilizing a monochromatized CuK radiation (λ = 1.54056 Å). UV-Vis spectra were accomplished utilizing a Shimadzu UV-2550. High-performance liquid chromatography (HPLC) was supplied on an Agilent 1260 Boundlessness instrument equipped with Wartz XTerraRP18 chromatographic columns with a length of 150 mm and inner diameter of 4.60 mm to determine the content of doxepin-loaded NiO NPs.

### 2.2 Preparation of NiO NPs

After being separated, the *Cressa nudicaulis* plant was thoroughly cleaned with distilled water. After that, it was ground with a mortar and pestle and dried. Weighed plant cuttings (5.0 g) were combined with 100 mL of distilled water. Thereafter, the mixture was stirred for 2 h at 55 °C, and the extract was filtered and stored at 5 °C. The extracted substance had a Brix degree of 2.31 (wt%). To make NiO NPs, 7.3 g of Ni(NO_3_)_2_·6H_2_O was dissolved in 50 mL water and stirred for 35 min. Then, the Ni(NO_3_)_2_·6H_2_O solution was slowly diluted with 30 mL of the plant extract. For 5 h, the solution was stirred at 70 °C to generate a green gel. To produce the surface modified NiO NPs, the remaining product was dried for 4 h in an oven at 50 °C. The sediments were then calcined for 2 h at 500 °C to produce a black powder of bare NiO.

### 2.3 Preparation of doxepin-loaded NiO NPs

A standard stock solution (1000 g mL^−1^) of the drug was made by dissolving 0.025 g of doxepin in 10 mL of distilled water. After that, KH_2_PO_4_ solution was used to dilute this solution to make the standard working solutions, which ranged from 5 to 500 g mL^−1^. Then, varying amounts of NiO NPs (0.002–0.04 g) were added to these standard solutions, and the resulting mixtures were stirred for the desired period of time, which ranged from 0.5 to 24 h.

### 2.4 Optimization of doxepin loading

At this point, we looked at how important parameters like reaction time, pH, and quantity of NiO NPs affect the process. The pH of four vials containing 5 mL of 0.01 M doxepin solution was altered by adding HCl (0.1 M) or NaOH (0.1 M) solutions in order to examine the effect of pH. After that, the solutions were stirred for 12 h, and the free drug concentration was determined by HPLC analysis of the supernatants after centrifugation. Using eqn (1), the drug loading efficiency (DL%) was determined.1
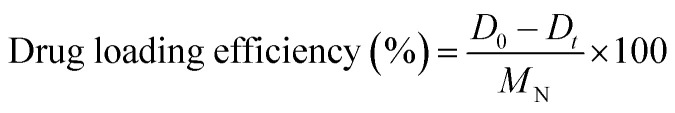
*D*_0_ and *D*_*t*_ represent the initial and free drug amounts, respectively. The amount of NiO nanoparticles is called *M*_N_. Doxepin (5 mL, 0.01 M) was added to four vials along with 0.02 g of NiO nanoparticles at pH 6. Then, the solutions were stirred for 0.5 to 24 h, the supernatants were separated, and HPLC was used to determine the peak area of each solution. The DL% was then determined using eqn (1). In the same way, the effect of NiO concentration was studied using nanoparticles (0.002–0.04 g) in 5 mL of a 0.01 M doxepin solution at pH 6 and stirred for 12 h. After centrifugation, the supernatants were separated, and the DL% was calculated in the same manner as before.

### 2.5 Studying *in vitro* drug release

The *in vitro* release of doxepin from NiO NPs was also achieved through the dialysis method. To release the loaded drug, 30 mg of drug-loaded nanoparticles was put into a dialysis bag (MWCO 1000 Da). Then, the bag was located in a 20 mL solution of PBS buffer at pH 6.5. The release system was then kept in the dark and slowly stirred in a constant temperature incubator shaker (50 rpm min^−1^, 37 °C). After that, 1 mL PBS buffer solution was frequently removed and replaced with a fresh medium. Then, the removed solutions were analyzed to determine the concentration of the released drug by monitoring the increment of the specific absorption band of doxepin at 497 nm by UV-Vis spectroscopy.

### 2.6 Cell culture and MTT assay

The MTT ((5,4-dimethylthiazole-2-yl)-5,2-diphenyltetrazolium bromide) test was utilized to determine the survival rate of the treated cell line at various NiO NPs concentrations (1000–5000 mg L^−1^). As a result, the Huh-7 cell line was used to test NiO NPs cytotoxicity. In the beginning, the cells were grown in Dulbecco's Modified Eagle Medium (DMEM), which contained penicillin and streptomycin as well as 10% fetal bovine serum (FBS). After that, the cells were kept at 37 °C in a CO_2_ atmosphere with 95% humidified air. 2 × 10^4^ cells were counted and placed in each well of 96-well plates involving 200 μL of culture medium for 24 h. The cells were then exposed to various concentrations of NiO to force their attachments to the bottom of the well. With DMSO serving as the control, 20 μL of the 5 μg mL^−1^ MTT solution was added to each well following incubation. After 4 h incubation, the culture medium was removed, and the MTT mixture in DMSO was poured into each well to dissolve the formazan crystals to determine the cell survival. At 570 nm, the absorbance of each well was determined with a plate reader. The percentage of cell viability against concentration (mg L^−1^) was then reported.^20^ Moreover, the amount of NiO nanoparticles that causes 50% of cell destruction was investigated in order to attain the inhibitory dose (IC_50_).

## 3 Results and discussion

### 3.1 Characterization of the fabricated NiO nanoparticles

The NiO NPs were identified using FT-IR, XRD, FESEM, EDS, TEM, and UV-Vis. The biocompatibility of NiO NPs and the ideal loading conditions were then investigated.

#### 3.1.1 FT-IR analysis

**Fig. 1:**
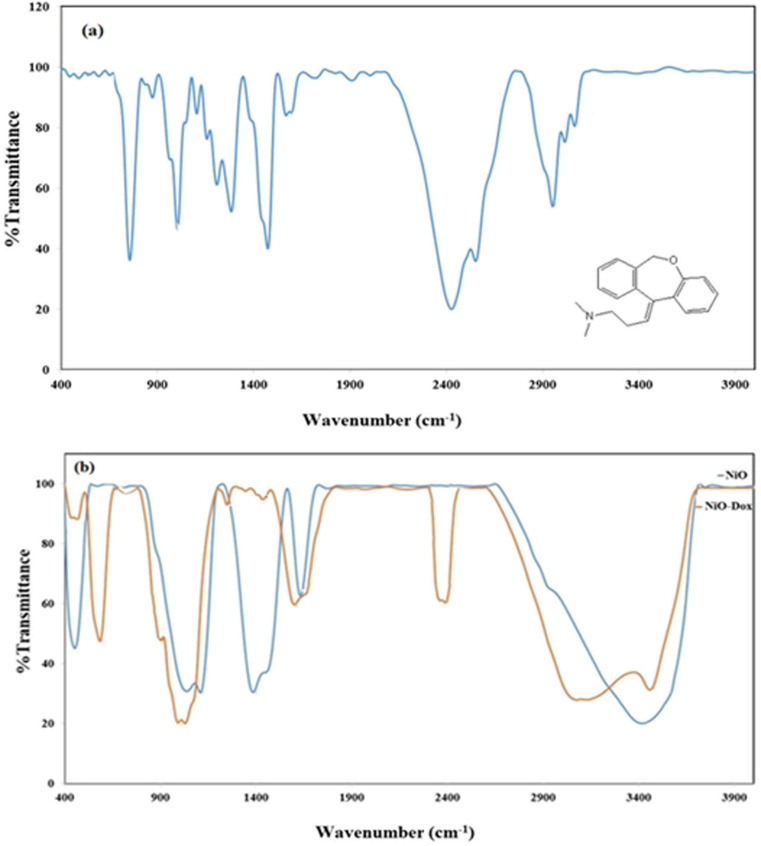
The FT-IR spectra of (a) doxepin and (b) NiO nanoparticles as well as doxepin-loaded NiO.

The FT-IR spectrum of doxepin and doxepin-loaded NiO NPs (NiO-Dox) are shown in Fig. 1. The N–H and C–H stretching vibrations due to doxepin were observed at 2940–3100 cm^−1^, respectively.^21,22^ The vibrational bands due to water molecules were also detected as a broad band around 3400 cm^−1^.^17,22–25^ After loading of doxepin, new peaks due to drug molecules were detected, as shown in Fig. 1b. Therefore, the FT-IR spectra confirmed loading of doxepin onto NiO nanoparticles.

#### 3.1.2 FESEM, TEM, EDS, XPS, UV-Vis, and XRD of NiO NPs

**Fig. 2:**
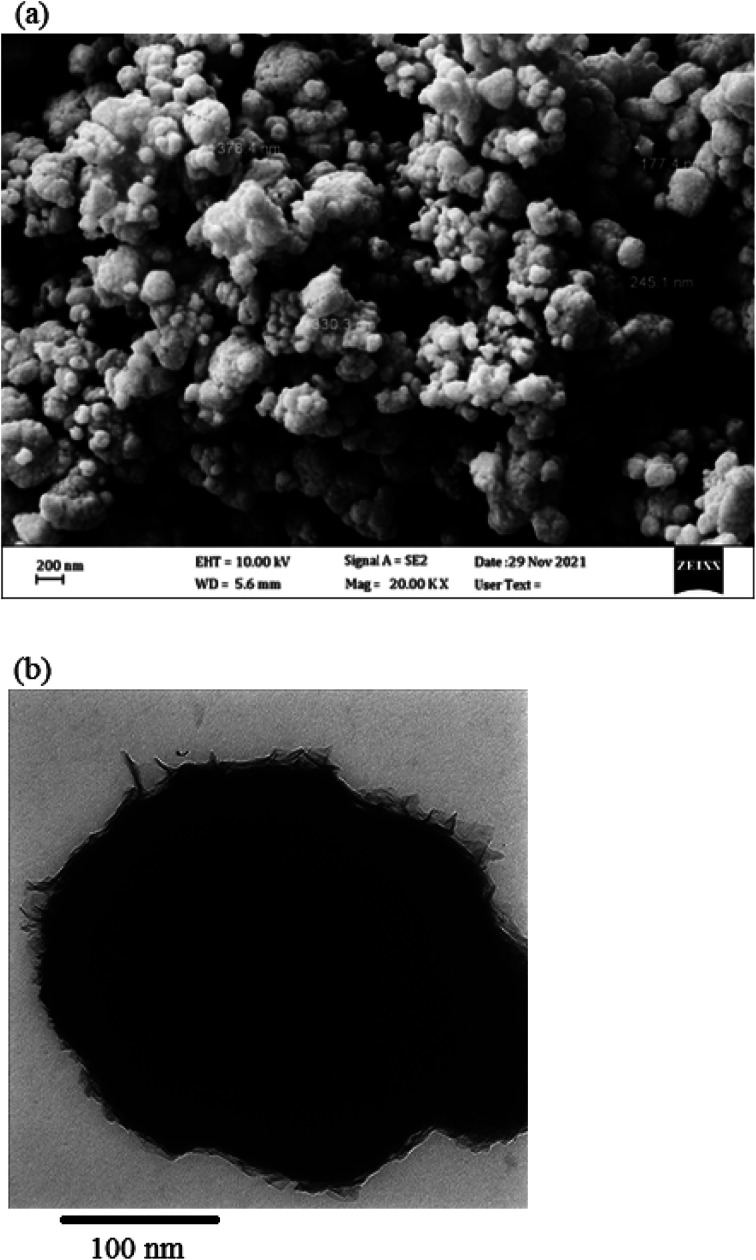
(a) FESEM and (b) TEM images of NiO nanoparticles.

**Fig. 3:**
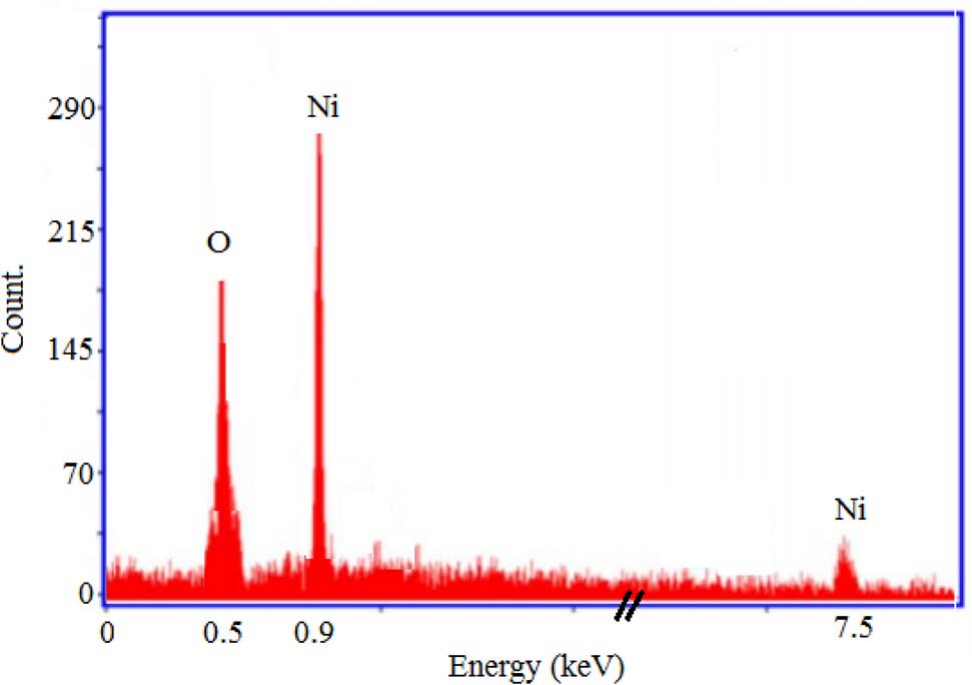
EDS analysis of NiO nanoparticles.

**Fig. 4:**
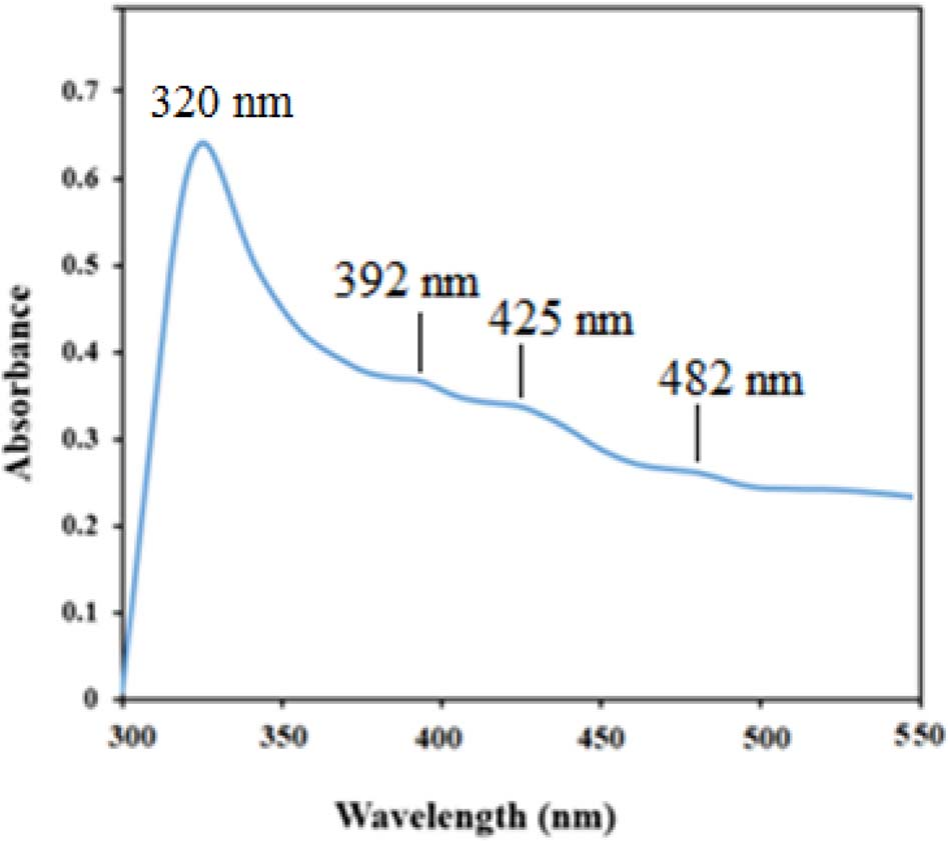
The UV-Vis spectrum of NiO nanoparticles.

**Fig. 5:**
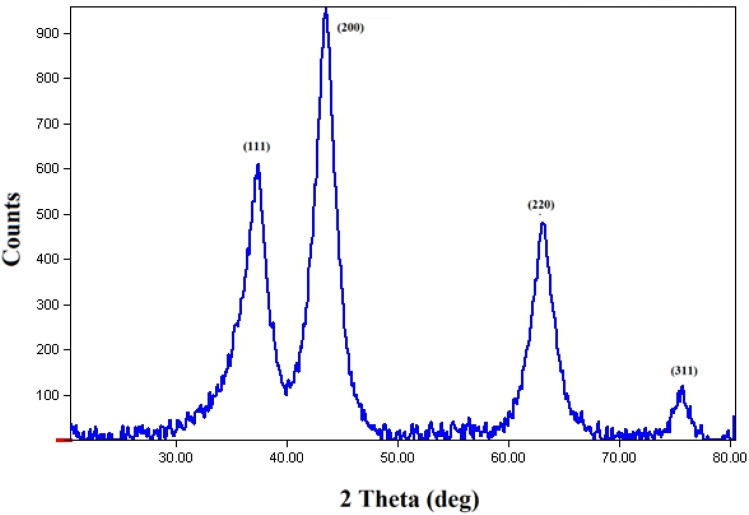
The XRD pattern of NiO nanoparticles.

The morphology and elemental composition of the prepared NiO NPs were revealed through FESEM and EDS analyses, respectively. The FESEM image revealed spherical particles with minimal agglomeration in some locations, and a mean particle size of 282 nm was proposed (Fig. 2a). Additionally, the TEM image of NiO nanoparticles revealed particles that were nearly spherical with approximately 250 nm in size (Fig. 2b). Using EDS, purity of the nanoparticles was confirmed, and it was found that NiO NPs are primarily composed of nickel and oxygen (Fig. 3). The UV-Vis spectrum of NiO NPs in the range of 300–550 nm is shown in Fig. 4. These nanoparticles have a broad ultraviolet absorption band at 320 nm. Other peaks at 392, 425, and 482 nm, which are caused by NiO surface plasmons, were also observed. The XRD pattern of NiO nanoparticles is shown in Fig. 5. In a cubic structure and the space group *Fm*3̄*m*, the observed diffraction peaks were due to (111), (200), (220), and (311) planes of NiO. The average crystallite size (*D*) of NiO nanoparticles was determined to be 258 nm from the largest diffraction peak (200) using the Scherrer equation (*D* = *Kλ*/*β* cos *θ*).

### 3.2 Drug loading efficiency

**Fig. 6:**
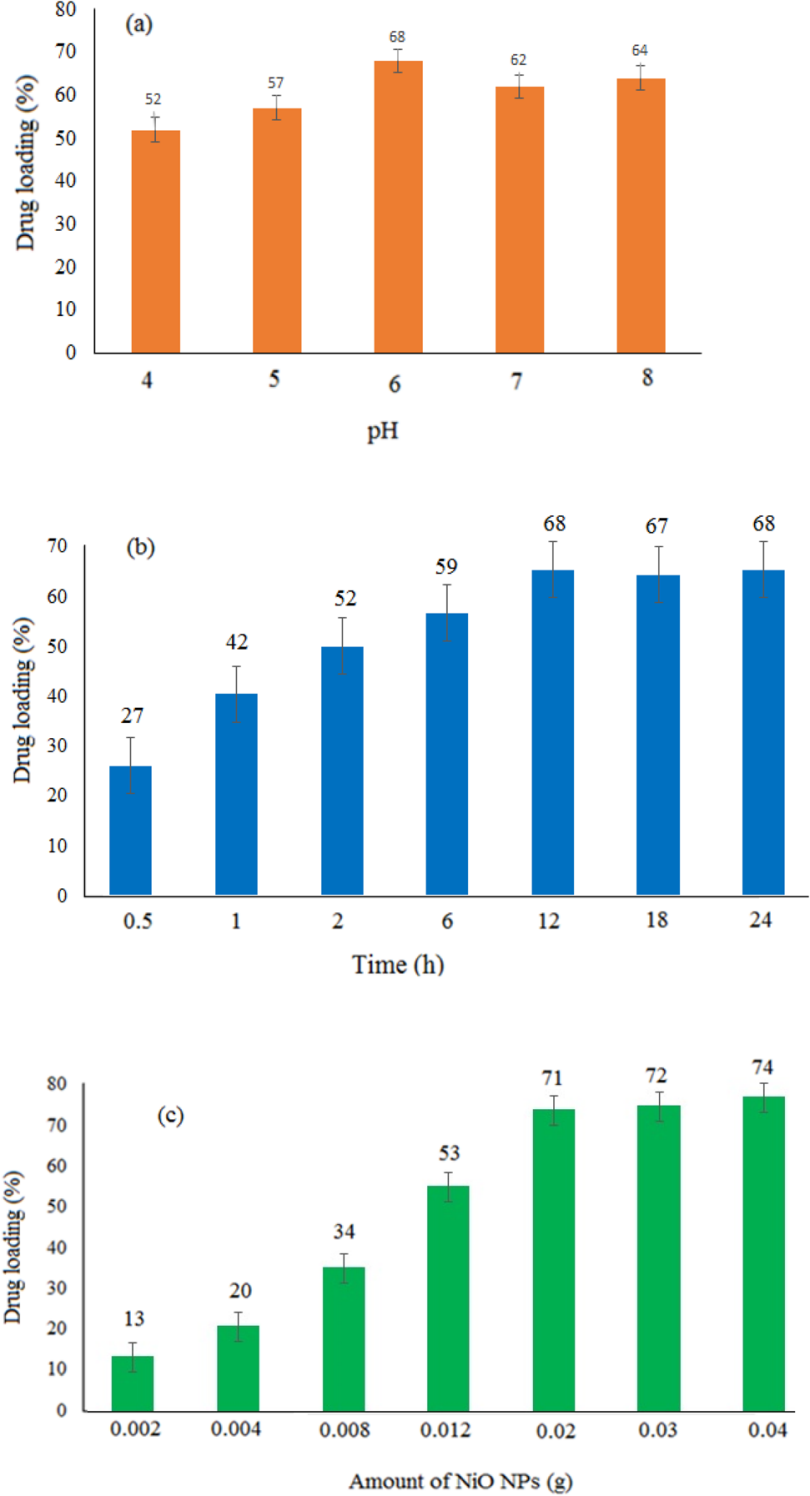
Drug loading (%) under three different conditions. Effect of (a) pH, (b) reaction time, and (c) amount of NiO NPs.

The effectiveness of the loading procedure would be evaluated by studying the capability of nanoparticles to load the targeted drug molecules. This study looked at the drug loading under different conditions involving pH, amount of nickel oxide nanoparticles, and reaction time. Adjusting the pH of the reaction medium was investigated to determine the best conditions for doxepin loading on the NiO nanoparticles. Since NiO precipitates at high pHs and doxepin is highly soluble in acidic pHs, this investigation was limited to pHs between 4 and 8. As seen in Fig. 6a, the highest drug loading (approximately 68%) was obtained at pH 6, and the drug loading was inclined as pH enhanced from 4 to 6. Under acidic conditions (pH 4), the drug molecules were protonated, reducing the formation of the H-bonds between NiO and the drug molecules, resulting in diminished drug loading. The effect of exposure time on the efficacy of loading for 0.5–24 h was also investigated. The interaction of drug molecules with nanoparticles was increased over time, and the highest drug loading was approximately 68% in 12 h (Fig. 6b). On the other hand, over a longer period of time (24 h), NiO may aggregate, and drug loading may decrease. Additionally, the effects of various amounts of NiO (0.002–0.04 g) on drug loading can be seen in Fig. 6c. Increasing the amount of NiO from 0.002 to 0.04 g resulted in an increase in drug loading from 13 to 74%. Due to the tendency of NiO for aggregation, the loading of drug molecules was decreased in the presence of higher amounts of NiO greater than 0.04 g. This would be due to the high surface area of nanoparticles, especially in the presence of higher amounts of nanoparticles. The best drug loading was obtained at pH 6, 0.02 g of NiO, and a reaction time of 12 h, with approximately 68% drug loading under the aforementioned optimal conditions.

**Fig. 7:**
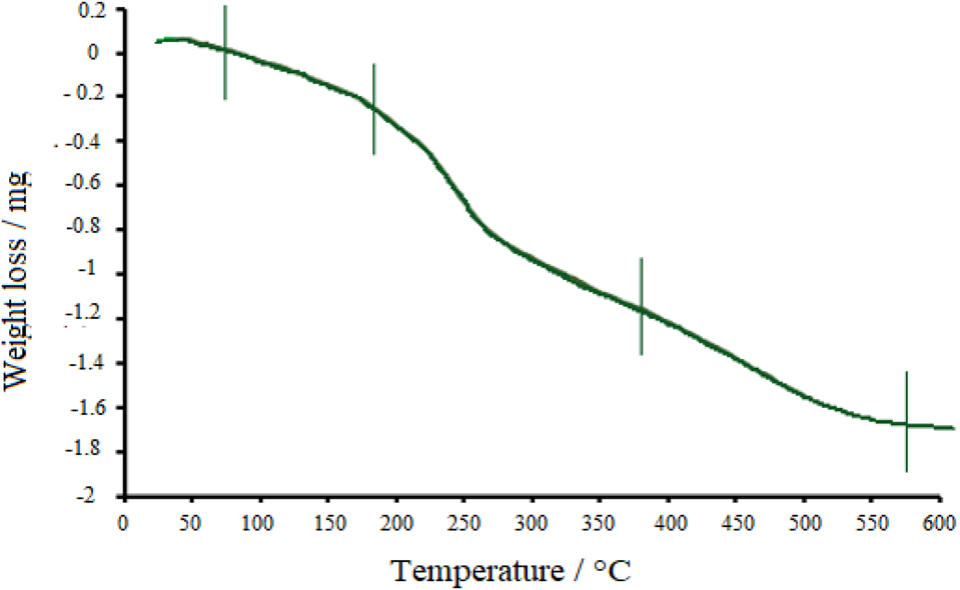
TGA profile of doxepin-loaded NiO NPs.

Thermal properties of the drug-loaded NiO nanoparticles were also explored by TGA (Fig. 7). Following the graph, three stages can be found in the decomposition process. The first weight-loss was observed below 180 °C, which is due to the desorption of water molecules from the surface of nanoparticles. Then, doxepin loaded onto NiO started to desorb, and drug destruction happened at about 380 °C, which further continued at higher temperatures. Finally, up to 500 °C, the crystallized NiO was attained.

**Fig. 8:**
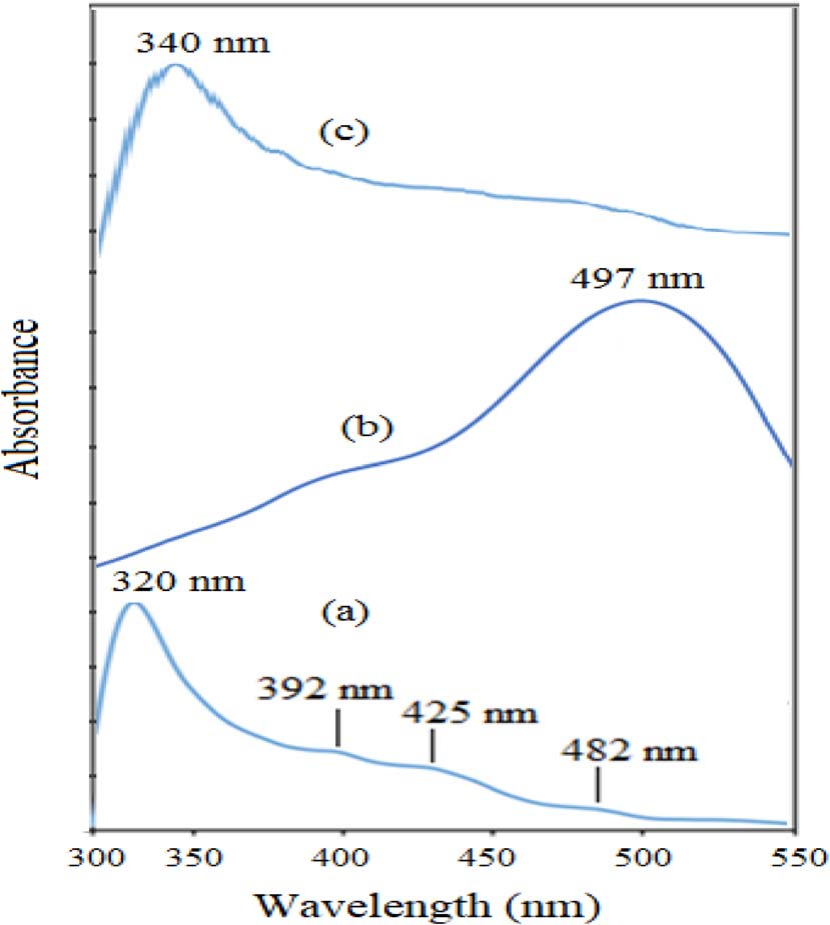
The UV-Vis spectra of (a) NiO nanoparticles, (b) doxepin, and (c) drug-loaded NiO NPs.

**Fig. 9:**
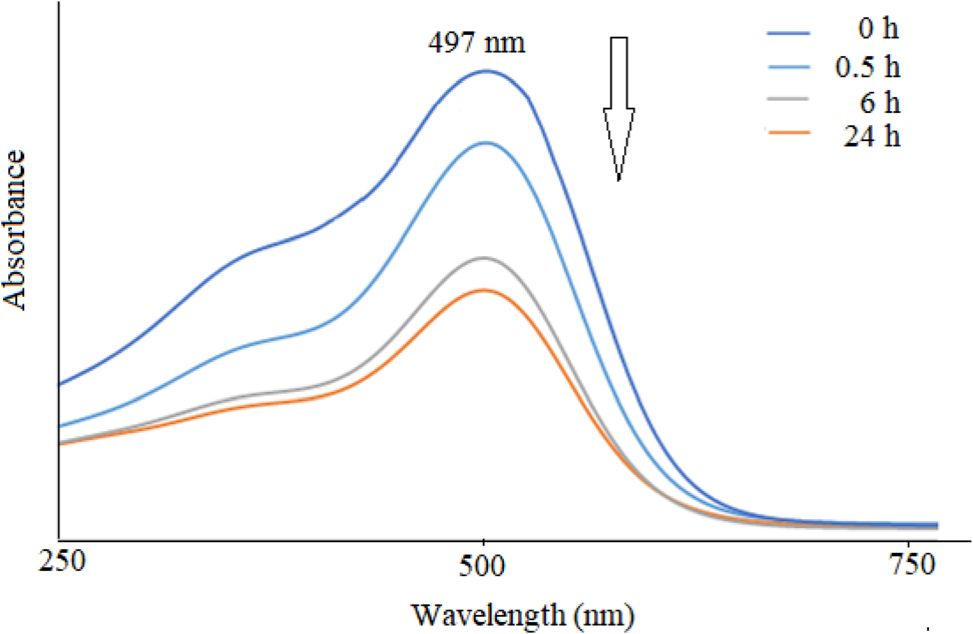
UV-Vis spectral changes of doxepin with time in the presence of NiO NPs.

The UV-Vis spectra of NiO nanoparticles, doxepin, and drug-loaded NiO NPs in the range of 300–550 nm are shown in Fig. 8. As previously mentioned, NiO NPs revealed a broad ultraviolet absorption band at 320 nm, which indicates a charge transfer from oxygen 2p (O^2−^) to 3d (Ni^2+^). When NiO NPs were combined with doxepin, the maximum absorption wavelength was red-shifted from 320 to 340 nm (Fig. 8c). This change might be attributed to the adsorption of doxepin from its protonated amino group to the negatively charged O^2−^ and hydroxyl groups on the surface of NiO NPs. Moreover, doxepin has a characteristic absorption peak at 497 nm. This broad band was monitored during drug loading, and it was inferred that an obvious decline in absorbance occurred after 24 h (Fig. 9).

**Scheme 1:**
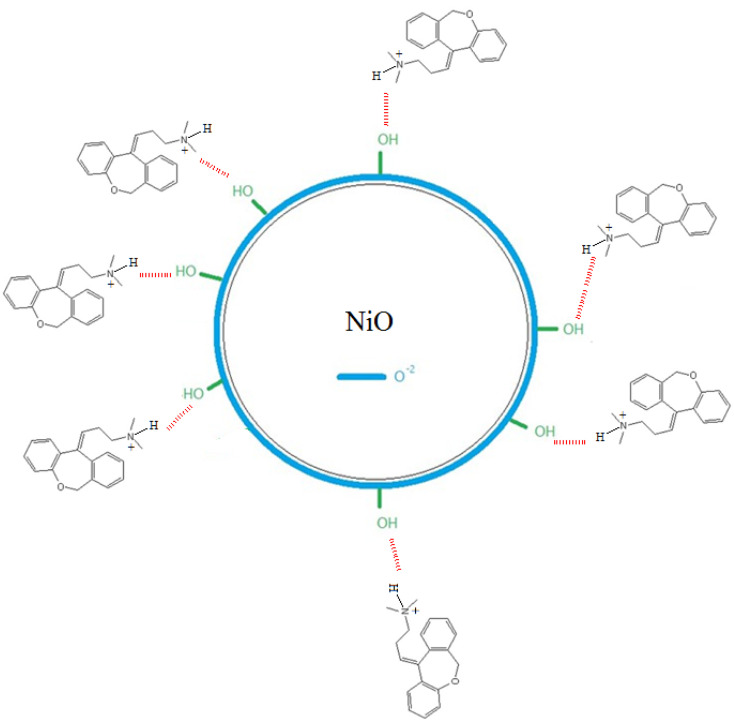
A schematic representation describing the interaction of doxepin with NiO nanoparticle.

Doxepin involves a protonated amino group under acidic conditions that contributes to the adsorption of the drug molecules onto NiO nanoparticles. According to Scheme 1, it can be inferred that the binding of doxepin to NiO NPs occurs *via* the electrostatic attractions between the protonated amine groups of doxepin with O^2−^ on the surface of NiO nanoparticles. In addition, hydrogen bonding between electronegative groups of doxepin and surface OH groups of NiO NPs facilitates the loading of the drug molecules onto nickel oxide nanoparticles.

### 3.3 A preliminary study on the *in vitro* drug release

**Fig. 10:**
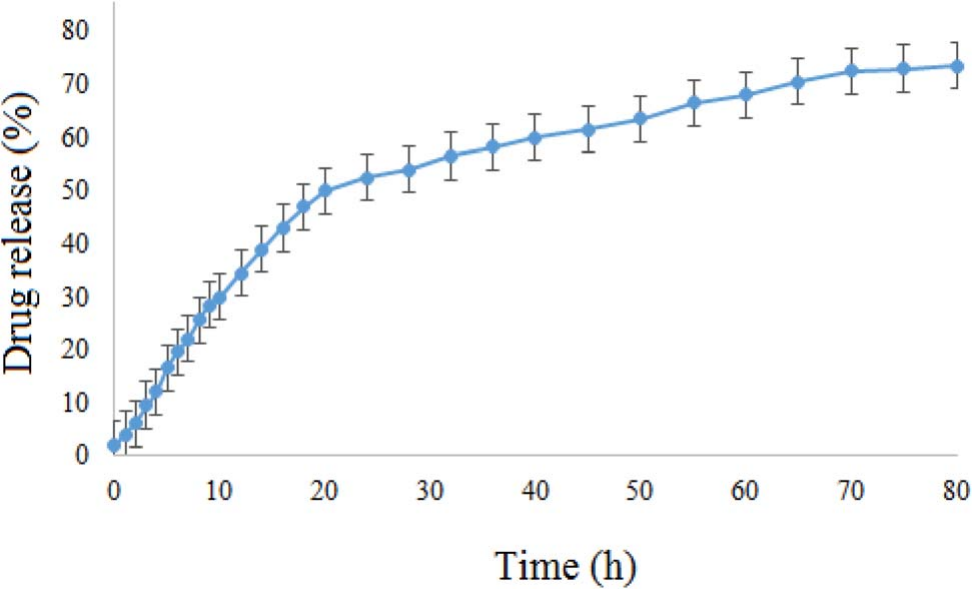
The *in vitro* release of doxepin from NiO-Dox at 37 °C in PBS buffer (pH 6.5).

The *in vitro* release of doxepin from NiO NPs was also investigated by means of the dialysis method. Since the pH of normal tissues in the body is about 7.4 and that of the tumor tissues is acidic (∼6.5), the *in vitro* release behavior of doxepin-loaded NiO was investigated using the dialysis membrane against potassium phosphate buffered (PBS, pH 6.5) solution at 37 °C (Fig. 10). This study showed that the drug-loaded NiO has a good releasing behavior and ∼73% of the loaded drug was released after 80 h. Therefore, NiO-Dox showed sufficiently long targeted-release properties and could improve the drug delivery process.

### 3.4 Cell culture and MTT assay

**Fig. 11:**
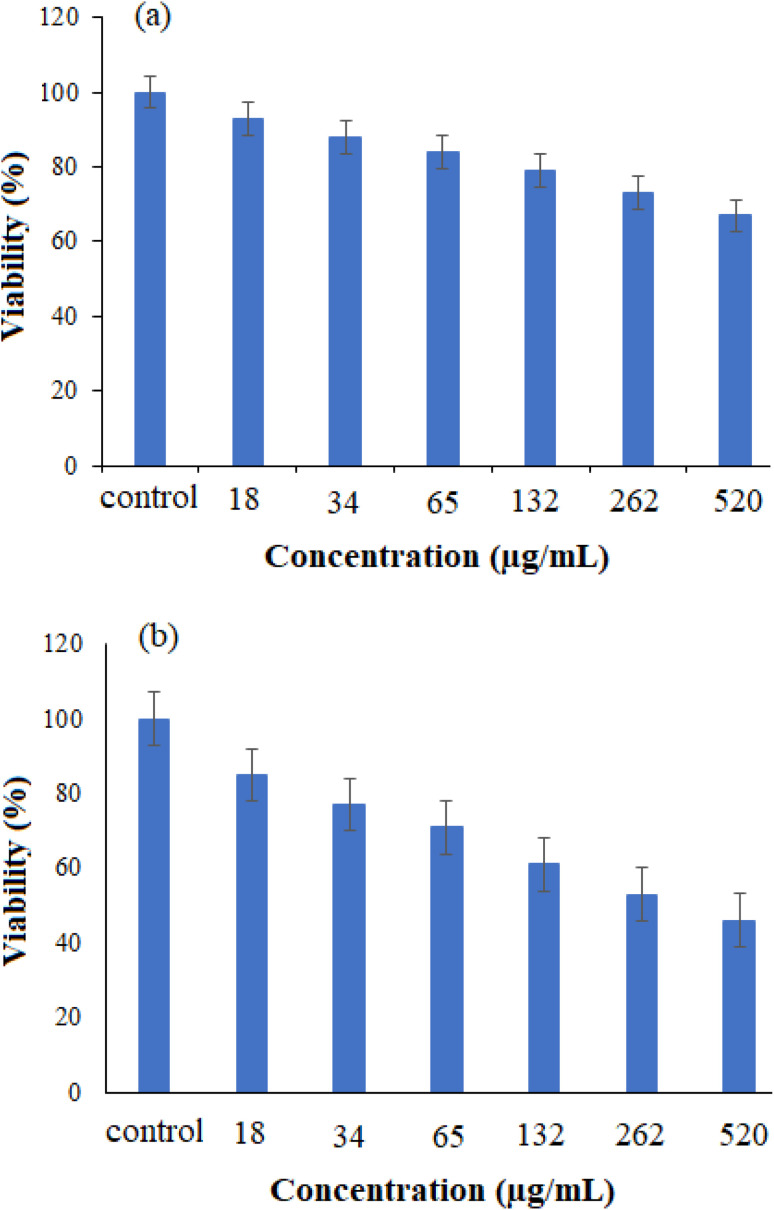
The cell viability of cancer Huh-7 cell line against (a) NiO NPs and (b) drug-loaded NiO NPs (*n* = 3, *P* < 0.001) using the MTT test.

The MTT assay was performed to test the cytotoxicity of bare (Fig. 11a) and drug-loaded (Fig. 11b) NiO nanoparticles on the cancer cell line Huh-7. Various metal oxide concentrations ranging from 18 to 520 μg mL^−1^ were used to determine the cell growth inhibitory concentration. As seen in Fig. 11, the drug-loaded NiO was preferentially more effective than the bare one in this experiment. This study demonstrated the dependency of cell-growth inhibition on the applied concentration. Therefore, the drug-loaded NiO NPs can be recommended as a potential candidate in cancer therapy.

## 4 Conclusion

NiO nanoparticles were synthesized from Ni(NO_3_)_2_·6H_2_O by the mediation of *Cressa nudicaulis* plant extract, and the fabricated nanoparticles were characterized by FT-IR, XRD, FESEM, EDS, TEM, and UV-Vis. Then, the doxepin drug was added to the nanoparticles to study loading capacity. After that, the pH of the reaction mixture, reaction time, and quantity of NiO nanoparticles were examined to see how they affected the drug loading efficacy. The best drug loading was found at pH 6 and 0.02 g NiO after 12 h, and approximately 68% of drug loading was attained under optimal conditions. The drug release properties of NiO nanoparticles were also investigated at pH 6.5 and 37 °C. This study showed that ∼73% of the loaded drug could be released after 80 h. Therefore, the introduced delivery system has sufficiently long targeted-release properties. In the final part, cytotoxicity of the bare and drug-loaded NiO NPs was investigated against the cancer cell line Huh-7. To the best of our knowledge, it is for the first time that NiO nanoparticles are prepared from *Cressa nudicaulis* under a green method. In addition, a brief literature survey showed that this work is the first to use green synthesized NiO nanoparticles to deliver doxepin.

## Data availability

Data supporting the findings of this study are provided throughout the text.

## Conflicts of interest

On behalf of all authors, the corresponding author states that there is no conflict of interest.

## Supplementary Material
